# Challenging a Benign, Elusive Tumor: Atypical Spinal Osteoblastomas in the Thoracic Spine with Surgical Resection and Hemi-Vertebral Body Reconstruction via a Posterior Approach—A Two-Case Series

**DOI:** 10.3390/reports9020152

**Published:** 2026-05-15

**Authors:** Joe Mehanna, Steffen-Heinrich Schulz, Sascha Gravius, Franz-Joseph Dally, Frederic Bludau

**Affiliations:** Orthopedic and Trauma Surgery Center, University Hospital Mannheim, 68167 Mannheim, Germany; steffen.schulz@umm.de (S.-H.S.); sascha.gravius@umm.de (S.G.); franz.dally@umm.de (F.-J.D.); frederic.bludau@umm.de (F.B.)

**Keywords:** osteoblastoma, thoracic spine, costotransversectomy, hemivertebrectomy, posterior spinal fusion, osteoid osteoma mimicry, spinal tumor

## Abstract

**Background and Clinical Significance:** Osteoblastomas are rare, benign but locally aggressive bone tumors with a predilection for the posterior elements of the spine. Their clinical, radiological and histopathological presentation often overlaps with that of osteoid osteomas, leading to diagnostic and therapeutic challenges—particularly in atypical locations such as the anterior thoracic spine. **Case Presentation:** We report two cases of young female patients (aged 35 and 30 years) presenting with persistent thoracic back pain unresponsive to NSAIDs. In the first case, imaging revealed a lesion at the right T7 pedicle initially attributed to osteoid osteoma; CT-guided thermoablation was declined due to proximity to neural structures. At this stage, we chose percutaneous transpedicular ablation by drilling through the centrum of the lesion (Nidus) surgically. After this transpedicular resection with initial symptom improvement, the patient developed recurrence with lesion progression into both anterior and posterior columns, requiring a second, open, surgical intervention. In the second case, a lesion at the left T11 pedicle and transverse process was identified directly as osteoblastoma due to size and radiological morphology; initial biopsy was non-diagnostic due to specimen fragmentation. In both cases, histopathology was inconclusive or misleading, while clinical and radiological features—including NSAID unresponsiveness, lesion size, and anatomical extent—favored osteoblastoma. Both patients underwent surgical resection via posterior costotransversectomy, partial hemivertebrectomy, expandable cage placement, and posterior instrumentation (T5–T8 and T10–T12, respectively). The postoperative courses were complicated by thoracic events—hemothorax in the first case and pulmonary embolism in the second—both of which were managed successfully. At follow-up, both patients were neurologically intact and pain-free. **Conclusions:** These cases emphasize the diagnostic overlap between osteoid osteoma and osteoblastoma and highlight the importance of clinical and radiographic correlation when histopathology is inconclusive. A posterior-only approach with costotransversectomy may be a valid strategy in selected cases of thoracic spinal tumors, although specific complications such as hemothorax must be considered.

## 1. Introduction and Clinical Significance

Osteoblastomas are benign yet locally aggressive osteoblastic tumors, accounting for approximately 1% of all primary bone tumors. They occur in the spine in about 10% of cases [1,2]. This type of tumor typically occurs in children and adolescents between the ages of 10 and 20 years, with a male predominance. More aggressive variants have been observed in patients around 30 years of age [3]. Osteoblastoma can develop in any bone; however, it shows a predilection for the spine, particularly the cervical and lumbar regions. Within the spine, it most commonly involves the posterior elements, especially the laminae and pedicles [2,4]. Patients typically present with back pain that is resistant to nonsteroidal anti-inflammatory drugs (NSAIDs) or aspirin. In some cases, the primary symptom may be a neurological deficit corresponding to the affected spinal level or a progressive spinal deformity, such as antalgic scoliosis [3]. In clinical practice, conventional spinal radiography plays an important role as an initial diagnostic tool, particularly in the presence of secondary deformities such as scoliosis or kyphosis. However, due to the complex anatomy of the spine and the high incidence of lesions in the posterior column, small or occult lesions may not be detectable on plain radiographs alone in the early stages [2]. Given that osteoblastomas can cause bone destruction, neurological symptoms, and exhibit fine ossification and calcification within the lesion, computed tomography (CT), in combination with magnetic resonance imaging (MRI) and positron emission tomography (PET), remains the primary modality for detecting the nidus and optimizing preoperative diagnosis and management [5]. More than 30% of spinal osteoblastomas show atypical radiological features, which may require CT-guided biopsy to confirm the diagnosis [6]. Left untreated, osteoblastomas may cause spinal instability, chronic and worsening pain, pathological fractures and neurological deficits. Surgical resection is therefore the treatment of choice, as these tumors are generally resistant to radiotherapy and chemotherapy [7]. In this report, we describe two unusual cases of spinal osteoblastoma affecting the thoracic spine in young female patients—one involving the right T7 vertebra with extension into both the anterior and posterior columns, and the other involving the left T11 pedicle, hemivertebra and transverse process. Due to the rarity of this spinal tumor, particularly in the thoracic region, a standardized surgical approach has not been well established in the literature. In this case series, we describe the surgical management, which in both cases involved costotransversectomy, partial hemivertebrectomy, expandable cage placement, and posterior spinal fusion.

## 2. Case Presentation

### 2.1. Case 1

A 35-year-old female presented with severe back pain unresponsive to nonsteroidal anti-inflammatory drugs (NSAIDs). At the time of presentation, there were no neurological deficits or signs of incontinence. Physical examination revealed localized tenderness to percussion over the thoracic spine. The family history of tumors was unremarkable. There were no clinical signs of scoliosis. Computed tomography (CT) and Magnet Resonance Imaging (MRI) demonstrated increased sclerosis at the posterior margin of the T7 vertebral body and within the right pedicle, where a nidus was identified. MRI demonstrated signal alterations surrounding the lesion on T1- and T2-weighted sequences, consistent with reactive inflammatory and bone marrow changes.

**Figure 1:**
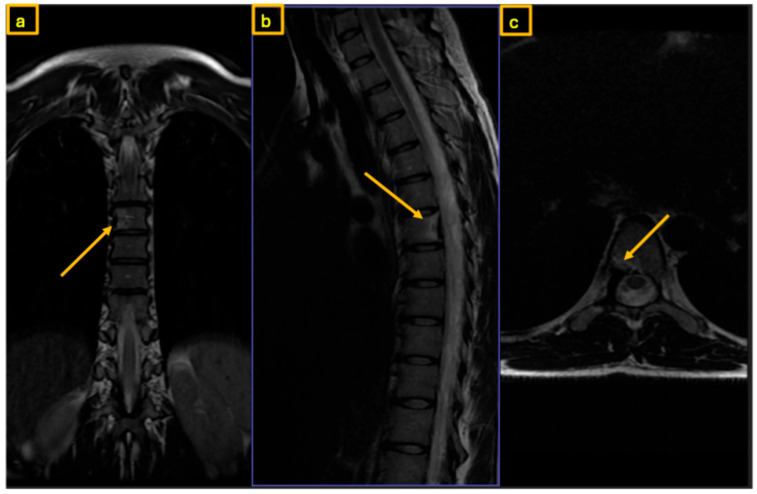
Preoperative MRI of the thoracic spine. The yellow arrow indicates the nidus at the root of the right T7 pedicle: (**a**) coronal view, (**b**) sagittal view, and (**c**) axial view.

**Figure 2:**
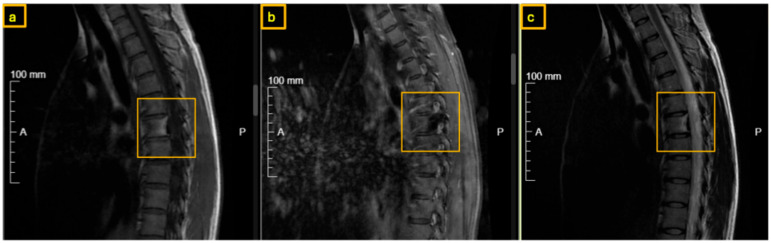
Preoperative MRI of the thoracic spine. The yellow square indicates the nidus at the root of the right T7 pedicle: (**a**) T1-weighted, (**b**) T1-weighted contrast-enhanced, and (**c**) T2-weighted sagittal views.

**Figure 3:**
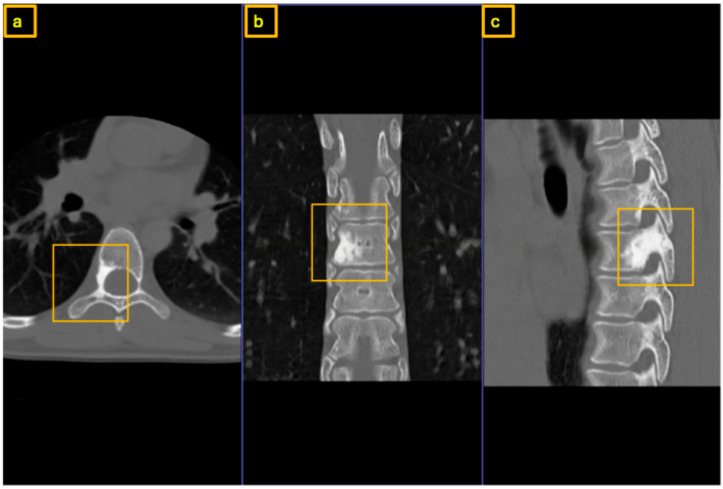
Preoperative CT of the thoracic spine for surgical planning: (**a**) axial view, (**b**) coronal view, and (**c**) sagittal view. The yellow square highlights the extent of the nidus within the posterior column, involving only the root of the right T7 pedicle.

Early sclerotic changes were also observed in the left pedicle (Figure 1, Figure 2 and Figure 3). The case was discussed with interventional radiologists regarding the feasibility of minimally invasive thermoablation. This was declined due to the high risk of neural damage in the neuroforamen and spinal canal. The differential diagnosis at that point included not only osteoid osteoma and osteoblastoma but also other spinal lesions such as osteoblastoma-like osteosarcoma, vertebral hemangioma, aneurysmal bone cyst, and atypical infections or metastases. Given the benign radiological features, absence of systemic signs, and small size of the lesion, malignancy was considered unlikely. A biopsy or resection was required to exclude more aggressive pathology.

**Figure 4:**
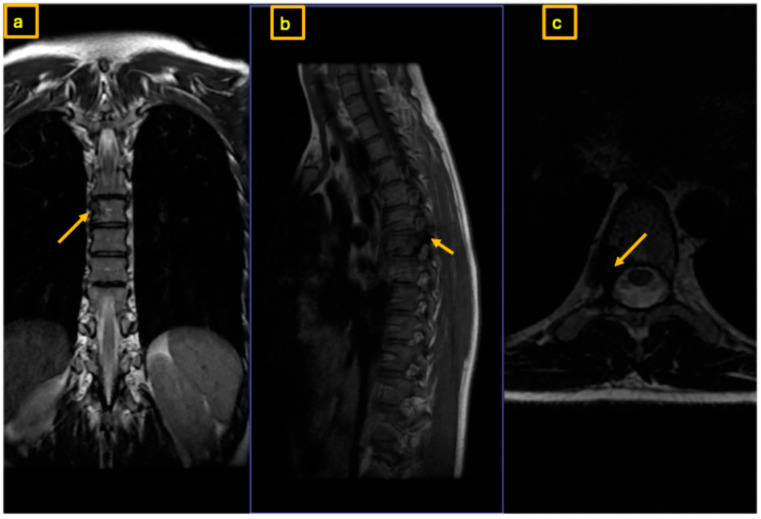
Postoperative MRI of the thoracic spine at 3-month follow-up. The yellow arrow indicates recurrence of the nidus at the root of the right T7 pedicle, with possible involvement of the vertebral body: (**a**) coronal view, (**b**) sagittal view, and (**c**) axial view.

**Figure 5:**
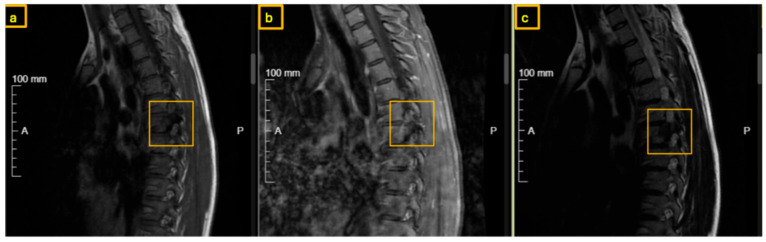
Postoperative MRI of the thoracic spine at 3-month follow-up. The yellow square indicates recurrence of the nidus at the root of the right T7 pedicle, with possible involvement of the vertebral body: (**a**) T1-weighted, (**b**) T1-weighted contrast-enhanced, and (**c**) T2-weighted sagittal views.

**Figure 6:**
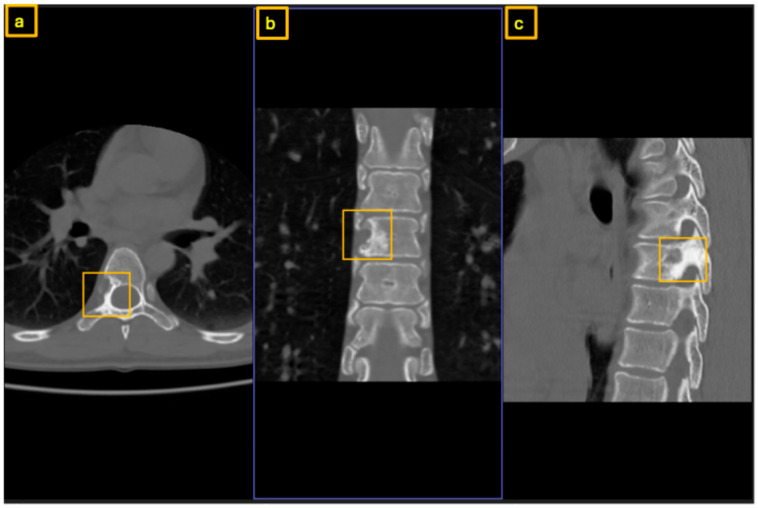
Postoperative CT scan at 3-month follow-up showing recurrence: (**a**) axial view, (**b**) coronal view, and (**c**) sagittal view. The yellow square highlights the recurrent nidus, now larger and infiltrating the right side of the T7 vertebral body.

Therefore, after concluding that osteoid-osteoma was the most likely diagnosis, we performed a surgical drilling and mechanical ablation via burr of the nidus on the root of the right T7 pedicle. Histopathological examination demonstrated osteoid-forming bone tissue with osteoblastic activity and without evidence of malignancy. Postoperative pain was significantly improved compared to preoperative levels. A few months later, the patient presented again with recurrent and increasing pain over T7, radiating cranially and caudally along the thoracic spine. During episodes of extreme pain, she experienced brief numbness in the left leg that did not correspond to a specific dermatome. An MRI was performed, showing normal representation of the spinal cord and surrounding cerebrospinal fluid. There was suspicion of residual or newly developed nidus and increasing sclerosis within the vertebra and lamina at the junction between the T7 vertebral arch and vertebral body on the right side (Figure 4 and Figure 5). The CT scan revealed a significant increase in sclerosis at T7, infiltrating the lamina, the remaining pedicle, and the vertebral body (Figure 6) well above the 1.5 cm threshold for osteoid-osteomas.

Given the strong suspicion of an osteoblastoma at T7 following resection of a nidus at the margin of the vertebral body and pedicle under the initial assumption of an osteoid osteoma, causing significant pain and reduction in quality of life according to the patient, there was a renewed indication for complete resection.

#### Surgical Technique

After minimal invasive ablation via drilling with a burr was deemed unsuccessful, we advised open resection and spinal fusion in order to avoid a transthoracic approach we opted for an all-posterior approach to which the patient agreed.

Following the team time-out protocol and intravenous antibiotic prophylaxis (Unacid 3g), a midline incision over T5–T8 was made. After subperiosteal exposure of the posterior elements, pedicle screws were inserted bilaterally at T5, T6, and T8 (Stryker K2M Everest^®^) under fluoroscopic guidance. A laminectomy at T7 with partial extension to T6 enabled decompression and access to the right costotransverse joint. The joint, the transverse process and part of the 7th rib were resected to expose the vertebral body of T7. The right T7 nerve root was identified, ligated, and resected to open up the path for hemivertebrectomy and implant placement. Local anesthesia (Carbostesin and Scandicain) was instilled into the nerve to prevent neuropathic pain. Under lung and anterior vascular protection using a smooth PSO (pedicle subtraction osteotomy) blade, using mainly small rongeurs, partial vertebral body resection of T7 was performed. Fittingly the bone was macroscopically as hard as stone from the osteoblastoma sclerosis. Resection was performed until there were clear margins of these sclerotic areas. Because the bone was highly sclerotic, the vertebra, pedicle and lamina had to be resected using a piecemeal technique as no whole tumor conglomerate resection was feasible.

Histopathological samples were collected. Intervertebral disks above and below were removed. An expandable vertebral body replacement implant (Signus Poseidon ST^®^ size D) with two 12 × 18 mm endplates (0°) was inserted via curved instruments and distracted to final position under fluoroscopic control. Irrigation and leakage check for pleural fistulas was performed via elevation of the PEEP (positive end-expiratory pressure) ventilation. A small pleural puncture was deemed minimally and conservatively manageable.

**Figure 7:**
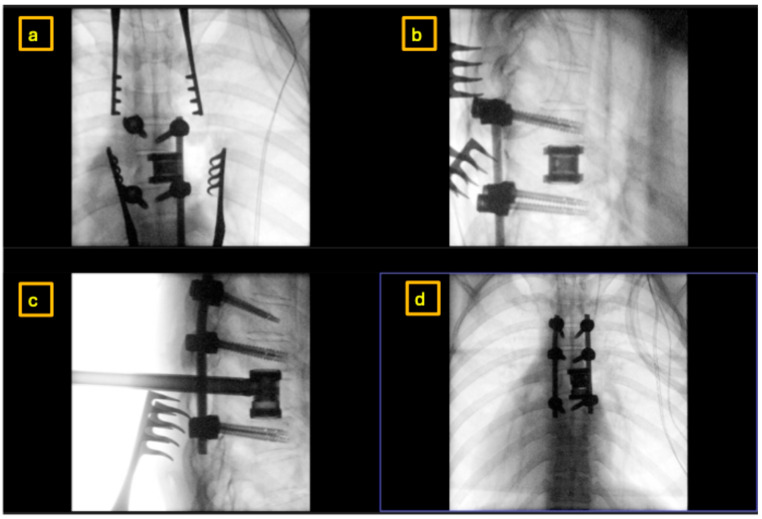
Intraoperative X-Rays showing placement of the expandable cage at the T7 level from the right via a posterolateral approach: (**a**,**d**) anteroposterior views; (**b**,**c**) lateral (sagittal) views.

Bilateral titanium rods were placed with right-sided caudal compression to correct mild scoliotic tilt. Posterior decortication coupled with autologous spongiosa placement was performed to induce posterior fusion. The final construct achieved posterior spinal fusion from T5 to T8 with decompression, hemivertebrectomy, stabilization, and anterior column support. Following thorough irrigation and hemostasis, final intraoperative imaging confirmed good implant positioning (Figure 7). The wound was closed in layers with intracutaneous suturing. Postoperatively, spontaneous motor activity was present. The surgery was performed without intraoperative neuromonitoring (IOM) as IOM can be a hinderance when trying to achieve tumor resection [8].

In the postoperative course, a right-sided hemothorax was detected leading to the indication for sterile placement of a chest drain, which was performed instantly after detection. The drain was removed 4 days later due to decreasing output volume and unremarkable fluid characteristics. Infection parameters, hemoglobin levels, and kidney function were monitored over the course of the hospital stay but remained unalarming. After removal of the drain the lung was completely expanded in radiographic chest films. The patient was discharged after 9 days.

The histopathological assessment following the initial transpedicular drilling confirmed components consistent with osteoid osteoma. However, after recurrence and more extensive resection, histopathology of the intraoperative specimens from the second surgery did not yield a definitive diagnosis. According to the consulting pathologist, the specimens consisted predominantly of fragmented sclerotic bone tissue and osteoid-forming components; however, specimen fragmentation and limited tissue architecture precluded definitive classification as either osteoid osteoma or osteoblastoma. Despite this limitation, the progression pattern, anatomical extent, intraoperative findings, and radiographic features—including lesion size, cortical destruction, hemivertebra and pedicle involvement—were strongly indicative of a spinal osteoblastoma. The diagnosis was therefore made based on the totality of the clinical, radiological, and surgical findings.

**Figure 8:**
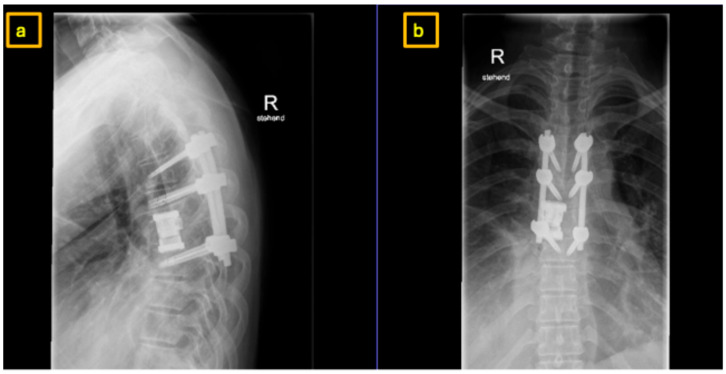
Postoperative X-Ray at 3-month follow-up demonstrating hemi-vertebral body replacement at T7 right and posterior spinal fusion from T5 to T8: (**a**) sagittal view, (**b**) coronal view.

**Figure 9:**
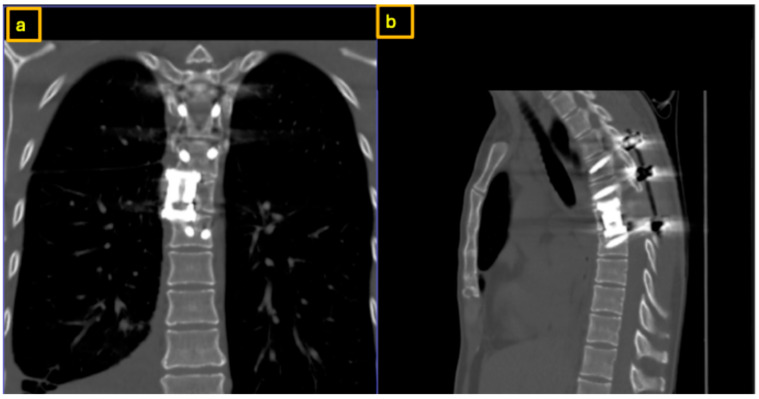
Postoperative CT-Scan at 3-month follow-up demonstrating hemi-vertebral body replacement at T7 right and posterior spinal fusion from T5 to T8: (**a**) coronal view, (**b**) sagittal view.

Three months and one year postoperatively, the patient presented for follow-up. Clinical findings were unremarkable (Figure 8). CT imaging demonstrated stable implant positioning without evidence of dislocation or material failure (Figure 9). The patient remained neurologically intact. Further annual follow-up was scheduled.

### 2.2. Case 2

A 30-year-old female presented with persistent thoracic back pain that had gradually progressed over several months, was exacerbated by physical activity, and was unresponsive to nonsteroidal anti-inflammatory drugs (NSAIDs). The pain was localized to the thoracic spine and increasingly interfered with her daily activities as well as her professional work as a mechanic. At the time of presentation, there were no neurological deficits or signs of incontinence. Physical examination revealed localized tenderness to percussion over the thoracic spine. Motor strength of the upper and lower extremities was preserved (Medical Research Council grade 5/5), and sensory function was intact. The family history of tumors was unremarkable.

**Figure 10:**
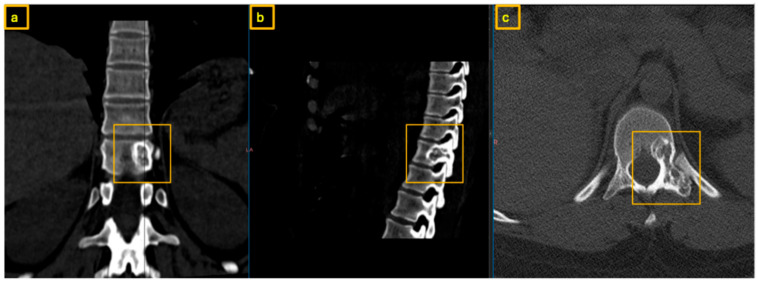
Preoperative CT of the thoracic spine. The yellow square shows the extent of the nidus within the posterior column, involving the root of the left T11 pedicle; (**a**) coronal view, (**b**) sagittal view, and (**c**) axial view.

Initial evaluation was performed at an external orthopedic institution. Computed tomography (CT) of the thoracic spine revealed a space-occupying lesion of uncertain etiology located within the left pedicle of the eleventh thoracic vertebra (T11) with extension into the transverse process with a small nidus present within the pedicle (Figure 10). Because the lesion demonstrated typical CT morphology without evidence of aggressive soft tissue extension or neurological compromise, additional MRI was not considered necessary for surgical decision-making. No clear radiologic signs of aggressive growth or malignancy were identified at that time. Given the persistence of symptoms and the unclear nature of the lesion, the patient was referred to our institution for further diagnostic evaluation and management.

An open surgical biopsy of the lesion at the left pedicle of T11 was performed. Histological analysis of the biopsy specimen demonstrated highly fragmented sclerotic and osteoid-forming bone tissue without evidence of malignancy; however, specimen fragmentation substantially limited definitive histopathological classification and histopathology was deemed inconclusive for osteoblastoma.

**Figure 11:**
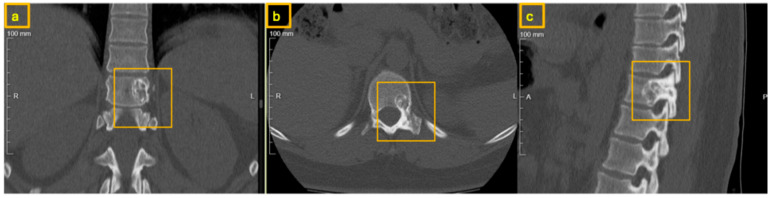
CT of the thoracic spine after the percutaneous biopsy. The yellow square highlights the extent of the nidus within the posterior column, involving the root of the left T11 pedicle, (**a**) coronal view, (**b**) axial view, and (**c**) sagittal view.

After multidisciplinary discussion, in view of the persistent NSAID-unresponsive symptoms, the indeterminate histological diagnosis, and the clinical and radiological features suggestive of osteoblastoma—including lesion size exceeding 1.5 cm and typical pedicle involvement reaching into the vertebra—the decision was made to proceed with definitive surgical resection combined with spinal reconstruction and stabilization; therefore, another CT examination was necessary for preoperative planning (Figure 11). A posterior-only approach was selected to permit complete resection and simultaneous anterior column reconstruction while avoiding the additional morbidity and potential blood loss associated with a combined anterior thoracic approach, which was particularly relevant given the patient’s chronic anemia and refusal of blood transfusion.

#### Surgical Technique

The surgical intervention was performed under general anesthesia with the patient in the prone position. Following the WHO time-out protocol and intravenous antibiotic prophylaxis, a midline posterior incision extending from T10 to T12 was made. After subperiosteal exposure of the posterior elements, pedicle screws were inserted bilaterally at T10 and T12 under fluoroscopic guidance. A left-sided costotransversectomy was performed with partial resection of the eleventh rib to facilitate adequate access to the lesion. The left T11 nerve root was identified, ligated, and transected to allow for hemivertebral resection and implant placement. Local anesthesia was instilled into the nerve to prevent neuropathic pain. A laminectomy of T11 was carried out. The pathological lesion was addressed through resection of the left pedicle and partial hemivertebrectomy of T11. The intervertebral disks above and below were removed. Resection margins were chosen macroscopically with enough distance to the sclerotic parts within the pedicle root and vertebra. An expandable vertebral body replacement implant was inserted under fluoroscopic control to reconstruct the anterior column. Posterior instrumentation was completed with bilateral titanium rods. Autologous bone graft from the resected rib, combined with a ceramic bone substitute was applied to induce posterior fusion.

In the postoperative course, immediate radiographs demonstrated correct positioning of the instrumentation with no evidence of implant failure. On postoperative day two, the patient developed thoracic pain and severe dyspnea. Hemothorax was ruled out, and a CT scan revealed a pulmonary embolism involving the middle lobe artery with early signs of right heart strain and pneumonic infiltrates in the left lower lobe. Anticoagulation was initiated with enoxaparin and transitioned to apixaban prior to discharge. Because the patient was a Jehovah’s Witness with chronic anemia and refusal of blood transfusion, initiation of therapeutic anticoagulation for pulmonary embolism required careful interdisciplinary risk–benefit assessment, as potential hemorrhagic complications could not be managed with transfusion support; nevertheless, the risk of thromboembolic progression and cardiopulmonary deterioration was considered higher. Hemoglobin levels, wound status, and hemodynamic parameters were therefore monitored closely during hospitalization, and no clinically relevant postoperative bleeding occurred. On postoperative day three, transient somnolence occurred; cranial CT showed no pathological findings, and the episode was attributed to postoperative anemia combined with opioid analgesia. The patient, a Jehovah’s Witness with chronic anemia, declined blood transfusions. Instead, treatment with iron, vitamin B12, and folic acid supplementation was initiated and was weaned successfully off the opioids. Mobilization progressed uneventfully. The surgical wound healed without complications. The patient was discharged in stable condition at POD (postoperative day) 8.

Final histopathological evaluation demonstrated irregular woven bone trabeculae with osteoblastic lining and sclerotic bone surrounded by loose stroma. Features were suggestive of osteoid osteoma, though reactive changes from the prior biopsy could not be excluded. No malignancy was identified. Despite inconclusive histopathology, the final diagnosis of osteoblastoma was supported by lesion size exceeding 1.5 cm, typical pedicle involvement with extension into the vertebral body and transverse process, progressive NSAID-unresponsive pain, and the absence of malignant features on imaging and histopathological assessment.

**Figure 12:**
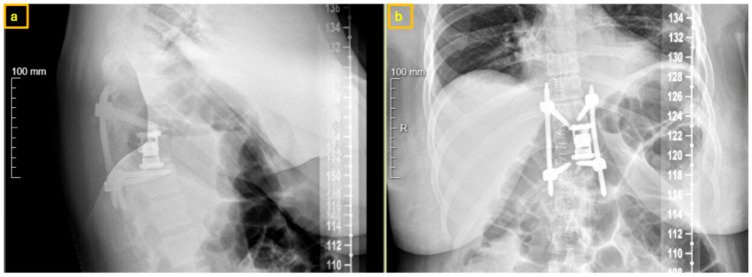
Postoperative X-Ray at 3-month follow-up demonstrating hemi-vertebral body replacement at T11 left and posterior spinal fusion from T10 to T12: (**a**) sagittal view, (**b**) coronal view.

At three- and six-month follow-up, the patient reported complete resolution of thoracic back pain and was able to return to normal daily activities without limitations. Neurological examination remained normal. Radiographic follow-up demonstrated stable instrumentation positioning without signs of implant loosening, displacement, or recurrence (Figure 12).

A summary of the clinical, radiological, surgical, and outcome characteristics of both cases is presented in Table 1.

## 3. Discussion

Not all small spinal lesions behave benignly. What appears radiologically as a classic osteoid osteoma may, over time, reveal a far more aggressive nature such as osteoblastoma. In Case 1, initial histology supported this benign diagnosis; however, the lesion recurred and expanded. Possible explanations include incomplete initial removal and underestimation of the lesion’s biological behavior, as osteoblastomas are known to demonstrate local progression and recurrence, particularly after limited intralesional treatment or incomplete resection [3,9]. In Case 2, the biopsy was non-diagnostic due to specimen fragmentation. Despite inconclusive histopathology in both patients, the clinical and radiographic course supported the diagnosis of osteoblastoma. This highlights the known difficulty in differentiating these two related tumors. While osteoid osteomas are typically <1.5 cm, present with a central nidus and respond well to NSAIDs, osteoblastomas usually exceed 1.5 cm in diameter, do not present with the classic history of pain suppressed by salicylates, behave more aggressively, do not regularly present with a nidus and tend to recur after incomplete resection [9,10]. Both of our patients presented with NSAID-unresponsive pain, lesions exceeding 1.5 cm, and typical pedicle involvement—features that collectively favor osteoblastoma over osteoid osteoma.

The diagnostic overlap between osteoid osteoma and osteoblastoma remains a significant clinical challenge. More than 30% of spinal osteoblastomas show atypical radiographic features, which may require CT-guided biopsy to confirm the diagnosis [6]. Consistent with previously reported spinal osteoblastomas, both of our cases demonstrated pedicle-centered lesions with surrounding sclerosis and extension into adjacent vertebral structures on CT imaging [2,5,6]. However, the progressive clinical course and inconclusive histopathological findings complicated definitive differentiation from osteoid osteoma.

In Case 1, the initial plan for CT-guided thermoablation was discussed with interventional radiology but was declined due to risk to adjacent neural structures. No biopsy was performed prior to surgery, as the lesion showed typical benign features. In Case 2, the biopsy yielded fragmented tissue without a definitive diagnosis. In both cases, the clinical decision to proceed with surgical resection was based on the totality of the clinical, radiological, and intraoperative findings rather than on histopathology alone.

In diagnostically uncertain cases, particularly when histological samples are inadequate, molecular testing for FOS (FBJ murine osteosarcoma viral oncogene homolog) and FOSB (FBJ murine osteosarcoma viral oncogene homolog B) rearrangements can offer additional diagnostic certainty. Fittall et al. demonstrated that rearrangements of FOS and FOSB are ubiquitous in osteoblastoma and osteoid osteoma, providing a molecular signature that distinguishes these tumors from malignant mimics [11]. FOS immunohistochemistry has emerged as a valuable ancillary diagnostic tool, with strong nuclear expression observed in 83% of osteoblastomas and 73% of osteoid osteomas [12]. Although molecular testing was not performed in our cases, this technique should be considered in future similar constellations where histology is inconclusive. Retrospective molecular analysis for FOS/FOSB rearrangements may provide additional diagnostic clarification in similar cases; however, in our patients, fragmented and limited specimen quality may restrict the feasibility and interpretability of such testing.

Following recurrence in Case 1 and persistent symptoms in Case 2, surgical intervention was indicated in both patients. The tumors had extended beyond the pedicle into the vertebral body, necessitating extensive resection. Both procedures were performed entirely via a posterior approach using costotransversectomy, which allowed hemi-corpectomy without the need for anterior thoracic access. This posterolateral route enabled decompression, resection of the infiltrated vertebral elements, and placement of an expandable vertebral body cage. Although combined anterior-posterior approaches are more commonly described for thoracic spinal tumors, posterior-only strategies have been successfully reported and offer the advantage of reduced morbidity in selected patients [13,14]. El Naga et al. demonstrated that nearly half of patients undergoing thoracic costotransversectomy experience an unanticipated short-term complication, and that this approach may be preferred in patients with significant medical comorbidity or tumors involving more than one thoracic vertebra [13].

To facilitate complete tumor removal, one-sided thoracic nerve root transection was necessary—the right T7 nerve root in Case 1 and the left T11 nerve root in Case 2. Neither patient developed neurological deficits besides numbness and paresthesia within a small area corresponding to the affected dermatome, which is consistent with reports indicating that thoracic rhizotomy is often well tolerated due to overlapping dermatomes and limited motor function [15,16]. Murakami et al. reported no neurological deterioration due to ligation of thoracic nerve roots in 79 patients undergoing total en bloc spondylectomy, confirming the safety of this approach in the thoracic spine [15].

Both patients developed postoperative complications related to the thoracic approach. Case 1 developed a right-sided hemothorax—likely related to rib resection and pleural manipulation—which was successfully managed with temporary chest drainage. Case 2 developed a pulmonary embolism on postoperative day two, requiring anticoagulation therapy. Such complications, though not uncommon, are known in thoracic spinal procedures and highlight the importance of early detection and multidisciplinary postoperative care [13,17], preoperative medical information, and inclusion of these specific complications in the consent forms.

Together, these two cases suggest a practical diagnostic and therapeutic approach for atypical thoracic osteoblastic lesions. Persistent NSAID-unresponsive pain, lesion size > 1.5 cm, pedicle involvement, progressive clinical course, and inconclusive biopsy findings should raise suspicion for osteoblastoma even when histopathology initially suggests osteoid osteoma. Both cases further demonstrate that posterior-only costotransversectomy with hemivertebrectomy, cage reconstruction, and posterior instrumentation can achieve successful resection and stabilization while avoiding an additional anterior thoracic approach, although thoracic perioperative complications must be anticipated and managed promptly.

This report has several limitations inherent to rare spinal tumor case series. Histopathological confirmation was partially limited by fragmented specimen quality and the overlap between osteoid osteoma and osteoblastoma. In addition, molecular analysis for FOS/FOSB rearrangements was not available, and long-term follow-up remains limited, particularly in Case 2. Furthermore, because of the rarity and heterogeneity of thoracic spinal osteoblastoma-like lesions, conclusions regarding optimal diagnostic and surgical management remain limited. Nevertheless, the combination of clinical presentation, radiological morphology, intraoperative findings, and postoperative course allowed meaningful diagnostic and therapeutic assessment of these unusual thoracic lesions.

Lastly, the demographic and anatomical characteristics of this case series are unusual. Osteoblastomas of the spine are rare overall, more frequent in males, and typically involve the cervical or lumbar regions. Both of our patients were young females (30 and 35 years old) with thoracic spine involvement—A presentation that is exceptional and adds further relevance to this report.

## 4. Conclusions

This case series highlights the diagnostic challenge posed by spinal osteoblastoma, where histopathology may be inconclusive and clinical–radiographic correlation becomes essential for diagnosis. When histology fails, molecular testing for FOS or FOSB rearrangements may provide diagnostic clarification. Surgically, posterior-only vertebral body reconstruction via costotransversectomy represents a safe and effective approach for thoracic spinal tumors, achieving complete resection and stable reconstruction while avoiding the morbidity with combined anterior-posterior procedures. However, these all-posterior-approaches for hemi vertebrectomy and tumor reconstruction can yield complications that need to be diagnosed and managed. We therefore advise all spinal tumor surgeries to be performed in a university clinic or specialized spine surgery center.

## Figures and Tables

**Table 1:** Comparative overview of the demographic characteristics, clinical presentation, radiological features, surgical treatment, histopathological findings, and postoperative outcomes of the two cases.

Parameter	Case 1	Case 2
**Age (years)/Sex**	35/Female	30/Female
**Symptom duration**	Several months	Several months
**Pain character**	Localized thoracic, radiating	Localized thoracic, activity-related
**NSAID response**	Unresponsive	Unresponsive
**Neurological status**	Intact (transient left leg numbness)	Intact
**Tumor location**	T7, right pedicle with vertebral body extension	T11, left pedicle with transverse process extension
**Columns involved**	Anterior and posterior	Posterior (with partial anterior)
**Lesion size**	>1.5 cm	>1.5 cm
**Initial intervention**	Transpedicular drilling/burr ablation	Open surgical biopsy
**Initial histology**	Osteoid osteoma	Inconclusive (fragmented specimen)
**Recurrence/Progression**	Yes	N/A (primary resection)
**Definitive surgical approach**	Posterior costotransversectomy	Posterior costotransversectomy
**Procedure**	Hemivertebrectomy, laminectomy, cage placement	Hemivertebrectomy, laminectomy, cage placement
**Instrumentation**	T5-T8 posterior fusion	T10-T12 posterior fusion
**Nerve root sacrifice**	T7 (right)	T11 (left)
**Final histology**	Inconclusive (fragmented specimen)	Suggestive of osteoid osteoma
**Clinical diagnosis**	Osteoblastoma	Osteoblastoma
**Postoperative complication**	Hemothorax (chest drain)	Pulmonary embolism (anticoagulation)
**Neurological outcome**	No deficits	No deficits
**Pain outcome**	Controlled	Complete resolution
**Follow-up**	12 months	6 months

## Data Availability

The original data presented in the study are included in the article, further inquiries can be directed to the corresponding author.
